# Stringhalt1Read before the annual meeting of the Pennsylvania State Veterinary Medical Association, at Philadelphia, March 6th, 1895.

**Published:** 1895-03

**Authors:** S. J. J. Harger


### STRINGHALT.[1]

BY S. J. J. HARGER, V.M.D.

It is not my intention to demonstrate in this paper the true pathology of this abnormal condition, and more careful observation is necessary before arriving at a definite conclusion. Here, let us remember that, as in many other cases of incorrect nomenclature, stringhalt, the "dry spavin" (*éparvin sec*) of the French, is only a symptom of diverse disease processes. The pathology of the disease or diseases with which this symptom is associated is not constant, and observation seems to demonstrate that there are a number of lesions in various parts of the member accompanied by altered muscular contractions which cannot be attributed exclusively to any one particular alteration. Hence, if we wish to study the pathology, it is, I believe, necessary to study a number of disease processes. In support of this view we have not only post-mortem dissections, but the variation in the group of muscles affected and the results of different methods of treatment; a treatment successful in one case may be negative in another.

The muscles affected are generally the flexors or the extensors in the different regions of the member. In some cases extension of the femur, flexion of the tibia and an excessive elevation of the point of the hock are most marked, without any exaggerated alteration in the canon movements; in others, there is excessive flexion of the femur and the canon, with an eleva-

[1] Read before the annual meeting of the Pennsylvania State Veterinary Medical Association, at Philadelphia, March 6th, 1895.

tion of the stifle. On account of the anatomical disposition of the flexor of the metatarus, flexion of the femur cannot take place without a corresponding movement of canon, although the latter seems in many cases relatively greater than the former. Abductive movements are infrequent. Each individual case offers a different physiological mechanism.

I will give a *résumé* of the various theories and the treatment of the defect, to be followed by a report of a case on which I operated with success. It was this latter fact which induced me to write this article.

Stringhalt is designated *idiopathic* when it cannot be assigned to any particular cause, and *symptomatic* or *mechanical* when it accompanies a definite lesion or a mechanical interference with locomotion.

Among these conditions I may mention pain in some portion of the member (symptomatic), curb, shortening of the fascia lata aponeurosis, spavin, a very straight hock, excessive development of the ridge of the lower articular surface of the tibia, shortening of the lateral extensor of the phalanges, chronic inflammation of the sciatic nerve. Goubaux observed chronic arthritis of the coxo-femoral joint. Pain in the foot under certain conditions acts as a cause. Cadiot made section successively of the digital and the sciatic nerves; in six days the spasmodic movement had disappeared, but fifteen days later the foot sloughed off. The hoof showed a keraphocele. Beyond this I am not prepared to speak on the foot theory of stringhalt. A hypodermatic injection of cocaine over the plantar nerves will eliminate or affirm the location of the cause in the foot. Again, in incipient spavin, before the exostosis is developed, a jerking movement of the hock may accompany the lameness. Undoubtedly in many such lesions are coincidents, while the true cause is not discovered.

Rousseau, a French army veterinarian, is of the opinion, after a number of observations, that the spinal cord is the seat of the lesion. He performed plantar and sciatic neurectomy to exclude the lower portion of the member, which did not influence the movements. Double stringhalt, arching of the back, tucked up flank and sensitive loins, yielding readily to the pressure of the hand, difficulty and exaggeration of the movements in turning led him to locate the trouble in the lumbar region of the spinal cord. He made one autopsy and found an abundance of arachnoid fluid, a yellowish coloration of the inferior (motor) nerve roots, while the superior were white, and some vascular congestion. The member otherwise was normal.

This theory will best explain many of the different phenomena observed and the variations in the muscular regions effected. The nerves emanating from the lumbo-sacral plexus originate in different parts of the spinal cord, and hence the altered muscular contraction in any given case may vary according to the portion of the cord affected. Comparing the flexors and the extensors, the altered movements may be developed under two conditions: excessive contraction of the flexors, or a loss of co-ordination between the flexors and the extensors. The first proposition explains itself. As to the second, during locomotion, the contractions of the flexors and extensors follow successively and harmoniously, first the one set and then the other. If this harmony is disturbed and if the one contracts before that of the others has ceased, the movements become antagonistic and irregular. Thus, if the flexors commence to contract before the extensor force is expended, the flexors must contract with additional force to overcome the latter, and thus impart a jerking movement to the leg. There is, as it were, an antagonism between the extending and flexing force. This will furnish a plausible explanation for the fact that the spasmodic contractions will disappear with exercise or become cured spontaneously after prolonged rest. Opposed to this, however, is the fact that the symptom sometimes disappears after a certain treatment influencing a region other than the central nervous system.

Comény's[1] theory is that stringhalt is due to an excessive dryness of the hock-joint from an insufficient synovial fluid. The dryness of the articulation, he claims, can be recognized in the exterior by the adherence of the skin to the surface of the bones; the skin is less movable; the fluctuation of the saphena vein less distinct; the skin on the outer and the inner side of the hollow of the hock is separated only by a thin layer of connective tissue, and in severe cases almost in contact with each other. He claims to have found this peculiarity in several hundred horses. It is more marked in severe cases and can be best seen by comparing the hock with that of the other leg or, in double stringhalt, with those of another horse. I have never been able to observe this conformation of the hock in such cases. The

[1] Rec. de. Med. Vet. Nov. 1894.

dryness of the articular surfaces and the increased friction explain, according to him, the "scraped" appearance of the articular pulley of the astragalus; an extra and spasmodic contraction is necessary to overcome the resistance which imparts a jerking motion to the foot. This is not yet confirmed by other observations. Comény ascribes the diminished synovial secretion, in a far-fetched manner, to alterations in the spinal cord. He recommends, as a verification of his view, an intra-articular injection of egg-albumin, which should give temporary relief; exercise increases the synovial fluid, and hence improvement after exercise.

A too straight hock, too open in front, open angles of the member sometimes give the movements a spasmodic character. This can be seen in the dissected hock-joint in which the flexion as well as extension after reaching a certain point are completed with a "snap" or jerk.

Boccar, Dickerhoof and Bassi called attention to the elasticity of the tibial aponeurosis, and have by its section cured a number of cases. I am inclined to the belief that this may act as a mechanical cause, acting like an india-rubber band extending from the stifle to the canon, for several reasons. (1) Dissections show that these aponeuroses contain yellow elastic fibres and appear elastic; (2) Section of the aponeurosis has given relief as well as cured such cases. In one case of double stringhalt much relief followed section of the aponeurosis and the lateral extensor in one member, while severing the anterior tibial nerve on the opposite member gave no relief, showing that the difference was not due to section of the lateral extensor tendon. This elastic apparatus comprises more than the tibial aponeurosis. It consists of a complete superficial envelope extending from the thigh to the canon. In the thigh we find internally the crural aponeurosis, in front the fascia lata, outwardly the gluteal aponeurosis; these are continuous below with the superficial layer of the tibial aponeurosis, which has a special termination in the form of a *triangular slip*, below the tarsus, on the anterior extensor tendon in the bend of the hock.

As to treatment, cauterization of the loins as well as electricity were unsuccessful.

I have in a few cases tried anterior tibial neurectomy, but always with negative results. Dr. J. C. Meyer reported[1] a suc-

[1] Read before the Ohio State Veterinary Medical Association.

cessful case of tibial aponeurectomy after section of the anterior tibial nerve was negative.

Dr. H. H. Choate, of Lewiston, Maine, and myself operated upon a most severe case of double stringhalt; in one member the tibial aponeurosis and lateral extensor were cut. A marked improvement followed and progressed until the animal was destroyed six weeks afterward. In the other leg the anterior tibial nerve was cut with no improvement.

Tenotomy of the lateral extensor without section of the aponeurosis, in my experience, has little influence upon the gait either in stringhalt or in the normal leg.

A case was brought to my clinic at the University Hospital which I will report in detail.

The subject, a draft horse, weighing about 1400 pounds, had a history of falling down an embankment a year ago, but had been affected with stringhalt prior to that time. The symptoms were most severe, rendering the animal *absolutely useless*. Walking was very difficult, and trotting was of the hop-skip-and-jump order. It was with the greatest difficulty that the horse was made to walk, the feet seemed as if pinned to the ground. Before starting on a walk the leg was forcibly flexed, the hoof touching the flank and maintained there for some seconds. The canons and pasterns were covered with scars from cuts of the opposite foot. In turning the body pivoted on the hind feet.

The operation was performed January 26th in the following manner: The patient being cast, a side-line was placed around the leg above the hock, and one at the lower end of the canon. After the usual antiseptic routine the skin was punctured two nches below the tarsus and over the tendon of the lateral extensor of the phalanges, care being taken not to injure the collateral artery of the canon. A long tenotome then being inserted, was passed toward the inner side between the skin and the triangular termination of the aponeurosis; the edge of the instrument was then turned toward the bone and the section completed by drawing it outward with some pressure, the side-lines being pulled in opposite directions to straighten the leg. The instrument was next passed between the tendon and the canon bone from behind to before, and the tendon cut upward. The separated ends of the aponeurosis and the tendon can be felt through the skin when the section is complete. After getting up there was no perceptible improvement. Some amelioration was noticed the next day, which continued steadily for a week, when the spasmodic movements were not much noticeable, and the animal practically was ready for work. At the present time the horse is practically cured. There is nothing abnormal in walking; trotting is regular, the action perhaps a trifle high. It is only in making a short turn that the flexion of the hock is a little exaggerated, but the animal is as serviceable as before. I have operated on several other cases, but unfortunately the animals were destroyed before the result could be determined. In one dissection in which the spinal cord was not examined nothing abnormal was found, excepting, as I thought, numerous elastic fibres in the above-mentioned aponeurosis covering the muscles; the synovia of the hock-joint was abundant, and the articular surfaces were not scraped, but showed a few spots of ulceration. In this case section of the lateral extensor tendon had no effect; the tibial aponeurosis was not cut.

To recapitulate, the spinal and the mechanical elastic theories, it appears to me, include the majority of these cases.

